# Sudden onset of parathyroid hormone-independent severe hypercalcemia from reversal of tumoral calcinosis in a dialysis patient

**DOI:** 10.1186/s12882-016-0355-y

**Published:** 2016-09-29

**Authors:** Fareed B. Kamar, Bikaramjit Mann, Gregory Kline

**Affiliations:** 1 Department of Medicine, Cumming School of Medicine, University of Calgary, Calgary, AB Canada; 2 Department of Critical Care Medicine, Cumming School of Medicine, University of Calgary, Calgary, AB Canada

**Keywords:** Hypercalcemia, Parathyroid hormone, Hyperparathyroidism, Tumoral calcinosis, Metastatic calcification, Chronic kidney disease, Peritoneal dialysis, Hemodialysis

## Abstract

**Background:**

Tumoral calcinosis is a rare manifestation of extraskeletal calcification, featuring large calcified cystic masses in the periarticular regions of large joints. In chronic kidney disease (CKD), this disorder is thought to evolve through a chronically elevated calcium-phosphorus solubility product leading to calcium precipitation in soft tissue. Treating tumoral calcinosis in these patients involves interventions to lower the calcium-phosphorus product such as reduction in vitamin D therapy and intensive hemodialysis regimens.

**Case Presentation:**

We report the case of a 54-year old woman with polycystic kidney disease on peritoneal dialysis with widespread tumoral calcinosis in the context of hypercalcemic tertiary hyperparathyroidism who had been on long-term alfacalcidol therapy. After withdrawal of the vitamin D analogue and initiation of daily hemodialysis, there was rapid dissolution of her tumoral calcium deposits with the abrupt onset of parathyroid hormone (PTH)-independent transient hypercalcemia that resolved once the soft tissue deposits disappeared.

**Conclusions:**

Resorption of soft tissue calcific deposits may result in transient parathyroid hormone (PTH)-independent hypercalcemia. In CKD patients, this hypercalcemia causes a decrease in the PTH level, distinguishing it from tertiary hyperparathyroidism, though PTH may not be totally suppressed, the way it is seen in PTH-independent hypercalcemia in non-CKD patients.

## Background

Chronic kidney disease (CKD) represents a state of disordered calcium and phosphate regulation that often leads to calcium salt deposition in soft tissue. This phenomenon of metastatic calcification may be widespread, involving blood vessels, visceral structures, and subcutaneous and cutaneous tissues [1]. On rare occasions, periarticular calcifications may grow into tumor-like masses, a manifestation recognized as tumoral calcinosis [2]. In CKD, an elevated serum calcium-phosphorus (Ca x P) product is likely the most important factor in the pathogenesis of tumoral calcinosis [3]. We present the case of a 54-year old woman with polycystic kidney disease on peritoneal dialysis (PD) who developed extensive tumoral calcinosis in the context of an elevated Ca x P product, hyperparathyroidism and long-term administration of a vitamin D analogue. A reversal of the factors favoring mineralization (vitamin D therapy and low phosphate clearance) allowed for dissolution of the calcium deposits, which we hypothesize caused transient, severe parathyroid hormone (PTH)-independent hypercalcemia.

## Case Presentation

A 54-year old woman with end-stage renal disease (ESRD) secondary to polycystic kidney disease who was on continuous cycling PD (CCPD) was admitted to hospital with a polycystic liver infection. Her CCPD prescription had been as follows: six cycles involving a total dialysate volume of 11,800 mL over 9 h plus one daytime exchange. All of the dialysate bags consisted of a Dianeal 1.5 % dextrose solution containing a calcium concentration of 1.25 mmol/L. A peritoneal equilibration test performed before hospital admission indicated a 24-h urine output of 1697 mL and a Kt/V of 2.72. For the past year, she had complained of progressive pain and immobility of the hips, with scant soft tissue calcium deposits seen on x-rays 1 year before admission. In hospital, she was found to have new small, palpable, and firm masses around her hips and cervical spine. She had been taking the vitamin D analogue alfacalcidol for years at a dose of 1.25 mcg orally daily, and was not on a phosphate binder. She had elevated serum calcium, phosphate and PTH levels (Table 1). Her calcitriol level was low at 26 pmol/L. A whole-body positron emission tomography-computed tomography (PET-CT) scan with fluorodeoxyglucose before her hospitalization illustrated widespread tumoral calcinosis, mainly around the large joints. Her alfacalcidol was suspended and she was put on a low-calcium and -phosphate diet before being admitted to hospital for treatment of her infected liver cysts.

**Table 1:** Trend in the patient’s calcium, phosphate, and PTH levels before and after cessation of alfacalcidol and switching to daily HD for treatment of her tumoral calcinosis

Serum biochemical marker	Three years before presentation (on PD)	At presentation before HD	One month after daily HD initiation	Two months after hospital discharge	Normal reference range
Calcium, mmol/L (mg/dL)^a^	2.5 (10.1)	2.7 (11)	3.4 (14)^b^	2.2 (8.8)	2.1–2.6 (8.4–10.4)
Phosphate, mmol/L (mg/dL)	1.5 (4.7)	2.0 (6.3)	1.3 (4.1)	1.4 (4.3)	0.8–1.5 (2.5–4.6)
Ca x P product, mmol^2^/L^2^ (mg^2^/dL^2^)	3.9 (48)	5.6 (69)	4.4 (55)	3.1 (38)	^c^
PTH, ng/L (pg/mL)	1062 (1062)	332 (332)	84 (84)	158 (158)	7–37 (7–37)

^a^These total calcium levels have been corrected for the serum albumin concentration

^b^The pre-discharge ionized calcium was also found to be elevated

^c^Professional guidelines have recommended a Ca x P product target of less than 4.4 mmol^2^/L^2^ (55 mg^2^/dL^2^) in stages 3 to 5 CKD [11]

**Fig. 1:**
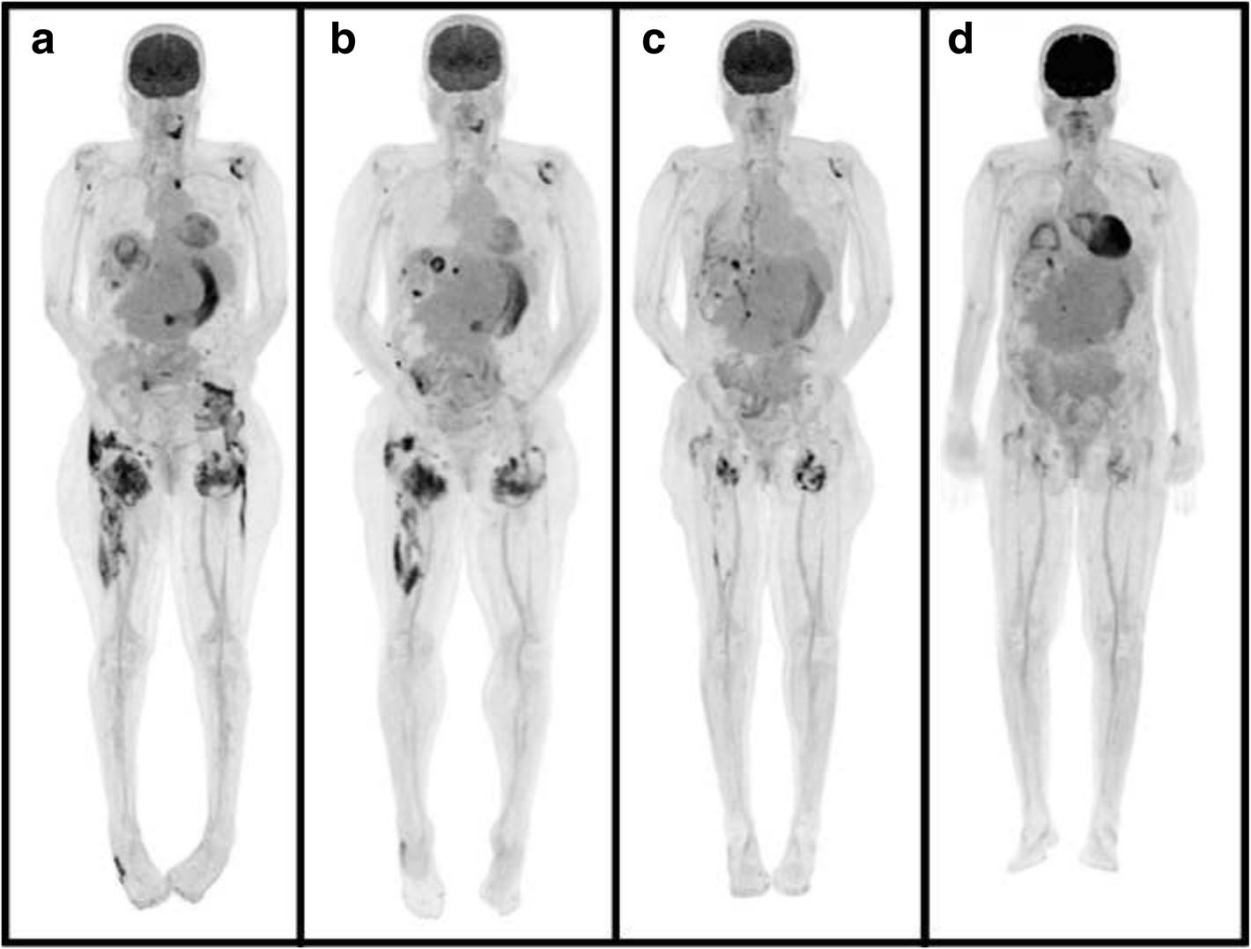
PET-CT images of the body illustrating tumoral calcinosis around the cervical spine, shoulders, left sternoclavicular joint, right buttock, hips, thighs, and right lateral midfoot at presentation **a**. These lesions dissipated after daily hemodialysis initiation in-hospital at 1 **b** and 2 **c** months later and at 2 months following hospital discharge **d**

Two months after the first PET-CT scan, a repeat PET-CT scan while in-hospital indicated progression of her tumoral calcinosis (Fig. 1a). For that reason, she was switched from PD to daily hemodialysis (HD). Her HD prescription involved 4-h sessions at a maximum blood pump speed with the following dialysate: sodium 138 mmol/L, potassium 4 mmol/L, calcium 1.25 mmol/L, and bicarbonate 28 mmol/L. Follow-up PET-CT scans one and 2 months later demonstrated significant improvement in her tumoral calcinosis (Fig. 1b, c) accompanied by a marked rise in serum calcium and a precipitous decrease in her chronically elevated PTH (Table 1). She was continued on daily HD after discharge from hospital following a 2-month admission. Two months later, these biochemical changes reversed with resolution of her tumoral calcinosis, as evidenced by the disappearance of the calcified masses on palpation and on PET-CT (Fig. 1d). She was then maintained on HD five times per week as she awaited an upcoming kidney transplant.

## Conclusions

CKD patients are predisposed to extraskeletal calcification because of an elevated Ca x P product [4]. In renal failure, phosphate excretion and vitamin D activation are impaired, causing hyperparathyroidism, which drives calcium release from bone [1]. Calcinosis often occurs, however, in the absence of hyperparathyroidism [2, 5], an observation that highlights the importance of PTH-independent ectopic mineralization-promoting factors in the generation of an elevated Ca x P product. Such factors that were present in this case included chronic hyperphosphatemia, long-term vitamin D analogue use, and possibly longstanding PD since residual renal function is known to decrease with time, occasionally with an insufficient compensatory increase in peritoneal phosphate clearance, especially in anuric patients [6].

Massive periarticular calcification, known as tumoral calcinosis, is a rare manifestation of extraskeletal calcification in CKD [2]. The mainstay of treatment involves measures to lower the Ca x P product [1]. In this case, a diet low in phosphate and calcium was administered and the vitamin D analogue was suspended, albeit without success. The switch from PD to daily HD [4] was, however, effective in treating the tumoral calcinosis.

It appeared that resolution of the patient’s soft tissue calcifications provoked transient but severe hypercalcemia. In CKD, the development of hypercalcemia is oftentimes the result of chronic vitamin D therapy with calcium-based phosphate binders. The patient’s increasing calcium level on HD occurred, however, after having stopped alfacalcidol. Prolonged stimulation of the parathyroid gland leading to autonomous PTH hypersecretion (tertiary hyperparathyroidism) is another cause of hypercalcemia among CKD patients [7]. Because the PTH in this case was “inappropriately high” for the severe hypercalcemia, tertiary hyperparathyroidism was considered in the differential diagnosis. However, a review of her chronic trends in PTH clearly showed that the calcium rise was acute and that the PTH of 84 ng/L was relatively “suppressed” in the context of pre-existing advanced parathyroid hyperplasia. A low PTH (i.e. < 100 ng/L [8]) in combination with hypercalcemia also prompted consideration of adynamic, or low-turnover, bone disease [9] although this entity too did not fit with the prior chronically elevated PTH levels and the acuity of the calcium and PTH changes.

Given the sudden PTH and vitamin D independence (fall in PTH and low calcitriol, respectively) of the hypercalcemia coinciding with the disappearance of the calcific masses clinically and radiographically, it was thought to be explained by exogenous calcium release from resorption of her tumoral calcinosis. Daily HD produced a drop in the Ca x P product, allowing for dissolution of the calcific lesions, causing soft tissue release of calcium into the intravascular compartment and hence hypercalcemia. This hypercalcemia caused relative PTH suppression instead of total suppression as would be expected in non-PTH mediated hypercalcemia outside of renal failure. Once the soft tissue deposits disappeared, the hypercalcemia and relative PTH suppression resolved, which was observed at 2 months following discharge from hospital. This phenomenon of tumoral calcinosis treatment possibly inducing hypercalcemia has been scarcely reported in the literature [2, 5, 10]. This case emphasizes the importance of an extensive review of the calcitropic hormone and mineral levels over time to assist with the interpretation of any apparent worsening of hypercalcemia in CKD. Additionally, this serves as a reminder that not all PTH-independent hypercalcemia necessarily requires the appearance of fully suppressed PTH levels, particularly in the context of CKD-associated chronic hyperparathyroidism.

Tumoral calcinosis is an unusual form of soft tissue calcification. When associated with CKD, measures should be taken towards lowering the Ca x P product and reducing the contributors to mineralization. As tumoral calcinosis resolves, release of soft tissue calcium into the general circulation may cause severe, relative PTH-independent hypercalcemia that is transient and may be observed without apparent harm to the patient, as was demonstrated in this case.

## Abbreviations

Ca x P: Calcium-phosphorus
CKD: Chronic kidney disease
ESRD: End-stage renal disease
HD: Hemodialysis
PD: Peritoneal dialysis
PET-CT: Positron emission tomography-computed tomography
PTH: Parathyroid hormone
